# Maternal Diabetes Alters Expression of MicroRNAs that Regulate Genes Critical for Neural Tube Development

**DOI:** 10.3389/fnmol.2017.00237

**Published:** 2017-07-27

**Authors:** Seshadri Ramya, Sukanya Shyamasundar, Boon Huat Bay, S. Thameem Dheen

**Affiliations:** Department of Anatomy, Yong Loo Lin School of Medicine, National University Health System, National University of Singapore Singapore, Singapore

**Keywords:** neural tube defects, neural stem cells, hyperglycemia, hypoglycemia, hypoxia, microRNA, microarray, miRNA-mRNA target genes

## Abstract

Maternal diabetes is known to cause neural tube defects (NTDs) in embryos and neuropsychological deficits in infants. Several metabolic pathways and a plethora of genes have been identified to be deregulated in developing brain of embryos by maternal diabetes, although the exact mechanism remains unknown. Recently, miRNAs have been shown to regulate genes involved in brain development and maturation. Therefore, we hypothesized that maternal diabetes alters the expression of miRNAs that regulate genes involved in biological pathways critical for neural tube development and closure during embryogenesis. To address this, high throughput miRNA expression profiling in neural stem cells (NSCs) isolated from the forebrain of embryos from normal or streptozotocin-induced diabetic pregnancy was carried out. It is known that maternal diabetes results in fetal hypoglycemia/hyperglycemia or hypoxia. Hence, NSCs from embryos of control pregnant mice were exposed to low or high glucose or hypoxia *in vitro*. miRNA pathway analysis revealed distinct deregulation of several biological pathways, including axon guidance pathway, which are critical for brain development in NSCs exposed to different treatments. Among the differentially expressed miRNAs, the miRNA-30 family members which are predicted to target genes involved in brain development was upregulated in NSCs from embryos of diabetic pregnancy when compared to control. miRNA-30b was found to be upregulated while its target gene Sirtuin 1 (*Sirt1*), as revealed by luciferase assay, was down regulated in NSCs from embryos of diabetic pregnancy. Further, overexpression of miRNA-30b in NSCs, resulted in decreased expression of Sirt1 protein, and altered the neuron/glia ratio. On the other hand, siRNA mediated knockdown of Sirt1 in NSCs promoted astrogenesis, indicating that miRNA-30b alters lineage specification via Sirt1. Overall, these results suggest that maternal diabetes alters the genes involved in neural tube formation via regulating miRNAs.

## Introduction

It is well-established that maternal diabetes is associated with neuroanatomical deficits (such as neural tube defects, NTDs) in the developing fetus (Jiang et al., 2008) and neuropsychological deficits in adult (Krakowiak et al., 2012). The development of the central nervous system (CNS), is controlled by various signaling molecules and transcription factors which regulate proliferation, differentiation, lineage specification, and migration of the neural stem cells (NSCs) (Umemori, 2014). NSCs, the progenitor cells of the CNS are self-renewing and multipotent giving rise to astrocytes, neurons, and oligodendrocytes (Gregg and Weiss, 2003). Although several studies attempted to understand the mechanism of hyperglycemia-induced brain malformations, the exact mechanism remains unknown. Recently, we have reported that altered epigenetic mechanisms (DNA methylation, histone modifications, and expression of miRNAs) may underlie hyperglycemia-induced malformations in embryos of diabetic pregnancy (Shyamasundar et al., 2013).

MicroRNAs (miRNAs) which are short, single-stranded non-coding RNA molecules approximately 22 nucleotides in length, with 70% of the known miRNAs expressed in different regions of the brain, have been shown to be critical for brain development and function (Lagos-Quintana et al., 2002; Cao et al., 2007; Kuss and Chen, 2008; Hoesel et al., 2010; Mukhopadhyay et al., 2011). They are sequence-specific post transcriptional regulators of gene expression (Lagos-Quintana et al., 2002). Maternal diabetes-induced NTDs have long been attributed to be caused by deregulation of several metabolic pathways, although the exact mechanism is still unclear. Given that gene expression profiling in cranial neural tubes of embryos from diabetic pregnancy has revealed altered gene expression in developing brains (Jiang et al., 2008), and that miRNAs regulate gene expression, we hypothesize that maternal diabetes alters the expression of miRNAs, which regulate genes critical for neural tube closure and patterning. To address this hypothesis, miRNA expression profiling using mouse NSCs isolated from forebrain of embryos from control and streptozotocin-induced diabetic pregnancy was performed. Since maternal diabetes has been shown to cause fetal hypoglycemia or hyperglycemia (Schwartz and Cornblath, 2000), NSCs isolated from embryos of control pregnancy were exposed to low or high glucose *in vitro* so as to mimic hypo- or hyperglycemia. Further, NSCs were cultured in reduced oxygen *in vitro* to mimic the hypoxic condition, as it has been shown that the excess glucose metabolism accelerates O_2_ consumption resulting in hypoxia in diabetic pregnancy (Li et al., 2005). The results obtained were compared in order to identify miRNA signatures implicating maternal diabetes-induced neural tube anomalies. Among the differentially expressed miRNAs in NSCs from diabetic pregnancy, the miRNA-30 family has been proposed to play critical role in maternal diabetes-induced neural tube anomalies as it has been shown to be involved in neurodevelopmental disorders (Mellios and Sur, 2012; Hancock et al., 2014; Sun et al., 2014; Han et al., 2015).

## Experimental procedures

### Animals

Seven to eight weeks old female ICR mice (InVivos, NUS) were made diabetic with a single intra-peritoneal injection of streptozotocin (STZ, 75 mg/kg body weight, Sigma-Aldrich, St. Louis, MO, USA), freshly prepared in 0.01 M citrate buffer (pH 4.5). A week later, the blood glucose levels of the mice were tested using a blood glucometer (Abbott's laboratories, Illinois, USA). Mice with non-fasting blood glucose levels of >200 mg/dl were considered diabetic, and were used for mating. Three to four diabetic female mice were caged with one healthy age-matched male mouse. The day that the copulation plug became apparent, was marked as embryonic day 0.5 (E0.5). In addition, time mated adult female pregnant ICR mice were procured from InVivos, NUS. At E13.5, the embryos from diabetic or control mice were collected through cesarean section, after anesthetizing them with pentobarbital (150 mg/kg body weight). The forebrains of the embryos were dissected and cultures of NSCs were set up. Only embryos from mothers with non-fasting blood glucose levels of >300 mg/dl were taken as experimental group. The procedures pertaining to animal usage was in accordance with the guidelines laid by Institutional Animal Care and Use Committee (IACUC), NUS.

### *In-vitro* culture of NSCs

The NSCs were isolated from the forebrain region of the embryonic brain. Briefly, the forebrains were dissected from the embryos and mechanically dissociated in 1X PBS solution. The dissociated cells were washed twice in 1X PBS and seeded in DMEM/F12 (1:1, ThermoFisher Scientific, Waltham, MA, USA). The cells were cultured in DMEM/F12 medium with 10 mM/L D-Glucose concentration, supplemented with insulin-transferrin-selenium (ThermoFisher Scientific), 20 ng/ml EGF (Sigma-Aldrich, St. Louis, MO, USA), and an antibiotic antimycotic solution (Sigma-Aldrich) in T-75 flasks (Greiner, Sigma-Aldrich). The cultures formed neurospheres that were maintained at 37°C/5% CO2 for 3–4 days, after which the supernatant containing the neurospheres was collected in 50 ml tubes (Greiner Bio-One GmBH, Germany) and centrifuged at 1,000 rpm/5 min. The supernatant was discarded and the cell pellets were dissociated with TryPLE™ Select (Gibco, Life technologies, Carlsbad, CA, USA) and re-plated into T-75 flasks. After 3–4 days the cultures were passaged again and used for different experiments. NSCs from normal pregnancy were cultured for 48 hours in medium with low glucose (2 mM D-glucose) or high glucose (40 mM D-glucose) concentration to mimic hypo- or hyperglycemia or in 1% oxygen for 4 hours to mimic hypoxia.

### Microarray profiling and analysis

Total RNA was isolated from NSCs from the different experimental groups using miRNeasy RNA isolation kit (Qiagen, Germany). Microarray profiling and data analysis were conducted at Exiqon services, Denmark. The quality of total RNA was first verified by Agilent 2100 Bioanalyser profile. Using the miRCURY LNA™ microRNA Hy3™/Hy5™ Power Labeling Kit, (Exiqon, Denmark), 750 ng of total RNA from sample (Hy3™ label), or reference (Hy5™ label) was labeled with a fluorescent label. Subsequently, the Hy3™-labeled samples and a Hy5™-labeled reference RNA samples were mixed pair-wise, and hybridized to the miRCURY LNA™ microRNA Array (6th gen—hsa, mmu & rno, miRBASE V16.0; Exiqon, Denmark). Hybridization was performed using a Tecan HS 4800™ hybridization station (Tecan, Austria), in accordance to the miRCURY LNA™ microRNA array instruction manual. Following hybridization, the miRCURY™ LNA array slides were scanned using the Agilent G2565BA Microarray Scanner System (Agilent Technologies, Inc., USA) and stored in an ozone free environment (ozone level below 2.0 ppb). The images were analyzed using the ImaGene 9.0 software (BioDiscovery, Inc., USA). The quantified signals were background corrected (Normexp with offset value 10, Ritchie et al., 2007) and normalized using the Quantile normalization algorithm. To identify the miRNAs that were statistically different between two NSC groups, a two tailed *t*-test was used, while one way ANOVA was used for more than two groups.

### Total RNA extraction

Total RNA (including small RNAs) was isolated from various experimental groups of NSCs using the Qiagen miRNEASY kit (Qiagen GmbH, Hilden, Germany), according to the manufacturer's instructions. The extracted total RNA was used for mRNA and miRNA qPCR analysis.

### cDNA synthesis and mRNA analysis

The cDNA synthesis was performed using 2 μg of RNA, 2 μl oligodT, 200 U of moloney murine leukemia virus (M-MLV) reverse transcriptase, 5 U of RNasin (Promega, Madison, WI, USA), and 2 mmol/L of dNTPs, in a 25 μl reaction volume. Firstly, 2 μg of RNA was mixed with 2 μl of oligodT and incubated at 70°C for 5 min in Bio-Rad™ T100 thermal cycler, following which the tubes were kept on ice for 1–2 min. Subsequently, 5X reaction buffer (5 μl), reverse transcriptase (1 μl), RNAase inhibitor (0.7 μl), dNTPs (0.5 μl), and nuclease free water, were added to the tubes and the cDNA synthesis was performed, by incubating the tubes at 42°C for 50 min, 95°C for 5 min and stored at 4°C. Gene expression was analyzed in Applied Biosystems (Model 7900 HT, Applied Biosystems, Foster City, CA, USA) using 10 μl reaction mix containing 5 μl SYBR green (Qiagen), 1 μM/L of each primer, 1 μl cDNA (from individual samples), and 3 μl nuclease free water in 96 well-FAST optical plates. Duplicates of each sample were loaded and an average was taken for further analysis. The 2^−ΔΔCt^ method was used to calculate the fold change in mRNA expression (Livak and Schmittgen, 2001).

### cDNA synthesis and miRNA analysis

To analyze miRNA expression, cDNA conversion of miRNA (from individual samples) was carried out using Universal cDNA synthesis kit II (Exiqon, Denmark). The expression of miRNA-30b and miRNA-30d were quantified by RT-PCR with Exilent SYBRGreen master mix (Exiqon) and microRNA primers for miRNA-30b or miRNA-30d (Exiqon) in 96 well-FAST optical plates (7900 HT, Applied Biosystems). Duplicates of each sample were loaded and an average was taken for further analysis. U6 snRNA (Exiqon) was used as the control. The 2^−ΔΔCt^ method was used to calculate the fold change in miRNA expression.

### Western blot

Total protein was extracted from NSCs using M-PER extraction reagent (ThermoScientific) and quantitated using the Bradford method (Bio-Rad, Hercules, CA, USA). Thirty micrograms of protein from each sample was denatured at 95°C for 5 min and loaded into 8 or 10% SDS-PAGE gel. Following electrophoresis, gels were blotted onto PVDF membranes and blocked in 5% non-fat milk for 1 h at room temperature. Primary antibodies were diluted in 5% milk and the PVDF membranes were incubated with the appropriate primary antibodies, rabbit anti-Sirt1 (ab12193, Abcam, Cambridge, United Kingdom) or mouse anti-Sirt1 (ab50517, Abcam), rabbit anti-Map2 (H-300, Santa Cruz Biotechnology, Dallas, Texas, USA), rabbit anti-Gfap (MAB 360, Millipore, Billerica, Massachusetts, United States), mouse anti-CNPase (MAB 326, Millipore), or mouse anti- beta actin (A1978, Sigma-Aldrich) overnight at 4°C. Subsequently, HRP-conjugated secondary antibodies [Anti-mouse HRP-31430; Anti-rabbit HRP-31460 (ThermoScientific)] were added and incubated for 1 h at room temperature. Chemiluminescence was detected using either pico or femto (ThermoScientific) substrates and signals were captured on X-ray films. The bands on the X-ray film was scanned and quantified using GS-800 calibrated densitometer (Bio-Rad, USA). Rabbit anti-Sirt1 was used for all experiments except to check Sirt1 silencing efficiency where mouse anti-Sirt1 was used.

### Transfection

NSCs from normal pregnancy were used to perform gain/loss-of-function studies. NSCs were trypsinized as mentioned previously and counted using the Bio-Rad TC 20 Automated cell counter, before seeding in 6-well-plates at a cell density of 1 × 10^6^ cells/well. Transfection was carried out using XTremeGene SiRNA transfection reagent, (Roche, Basel, Switzerland) for siRNA probes (Sirt1-4390771; negative control-4390843, Ambion, ThermoFisher Scientific) in Opti-MEM media at a final concentration of 50 nM. Transfection of miR-30b mimics (Ambion, ThermoFisher Scientific) and negative control probes (Ambion, ThermoFisher Scientific) were performed using lipofectamine RNAiMAX (ThermoFisher Scientific) in OptiMEM media at a final concentration of 10 nM. Opti-MEM medium was changed to NSC culturing media after 6 h post transfection. RNA was extracted at 48 h post transfection while protein extraction was performed 72 h post transfection.

### Luciferase assay using BV2 cells

Mouse microglial BV-2 cells were maintained in DMEM containing 10% fetal calf serum (FCS) at 37°C in a humidified incubator under 5% CO_2_. BV2 cells were co-transfected with miR-30b mimics or negative control mimics (at 10 nM) and plasmid vector containing luciferase gene and 3′UTR of Sirt1 gene (308 ng) (MmiT054166-MT06) using Lipofectamine® RNAiMAX (Thermofisher Scientific). The cells were cultured for 48 h after which luciferase activity was assayed according to the manufacturer's instructions using LUC-Pair Duo-Luciferase Assay kit 2.0 (LPFR-P010, Genecopoeia, Rockville, USA). The luminescence intensity was measured using a luminometer (Spectramax M5) and firefly luciferase activity was normalized to renilla luciferase activity.

### Immunostaining

The neurospheres were harvested and transferred to poly-L-lysine coated coverslips and poly-L-ornithine coated coverslips (the dissociated cells) and cultured in 24 well-plates for appropriate time points. The cells were fixed with ice cold 4% paraformaldehyde (PFA) for 30 min and the cells were permeabilized with phosphate buffered saline (PBS) containing 0.1% Triton-X 100 (PBS-T) for 20 min. Following that, the cells were blocked with 5% normal goat serum for 1 h before incubating with mouse anti-Sirt1antibody (1:500, ab50517, Abcam) overnight at 4°C. The next day, cells were incubated with Cy3-conjugated anti-mouse IgG secondary antibody (1:100, Chemicon, Temecula, CA, USA) for 1 h at room temperature. Finally, the nucleus was counterstained with DAPI and the coverslips were mounted on glass slides with fluorescent mounting medium (DAKO, USA). Images were captured with Olympus FV1000 confocal microscope.

### Pathway analysis

Pathway analysis using Ingenuity Pathway Analysis software (IPA, Qiagen) was performed to identify the mRNA targets of the differentially expressed microRNAs using expression levels of miRNAs in each of the NSC groups with a significant fold change of <−1.2 or >1.2 (*p* < 0.05) as input. IPA's microRNA target filter was applied to each dataset, and core analysis was performed to identify the canonical biological pathways. Comparison analysis was done to identify the common biological pathways among the different groups.

### Statistical analysis

At least three independent experiments were performed and the data was represented as mean ± SD. To identify the miRNAs that were statistically different between two NSC groups, a two-tailed *t-*test was used, while one way ANOVA was used for more than two groups. Student's *t*-test was done using Microsoft Excel spread sheet and data was considered significant when *p* < 0.05 (Control vs. Experimental, *n* = 3–4).

## Results

### Global miRNA expression profiling reveals distinct miRNA expression in NSCs exposed to high glucose

In order to determine whether maternal diabetes alters the expression of miRNAs in NSCs of the developing brain, global miRNA expression profiling was carried out in NSCs isolated from embryos of control and diabetic pregnant mice using miRCURY LNA™ miRNA Array (containing ~2,383 capture probes). Since maternal diabetes can cause fetal hyper/hypoglycemia (Schwartz and Cornblath, 2000) or hypoxia (Teramo et al., 2013), NSCs derived from control pregnancy were exposed to high glucose to mimic hyperglycemia (HG) or low glucose to mimic hypoglycemia (LG) or 1% oxygen to mimic hypoxia (OX) *in vitro* and used for the microarray analysis.

**Figure 1:**
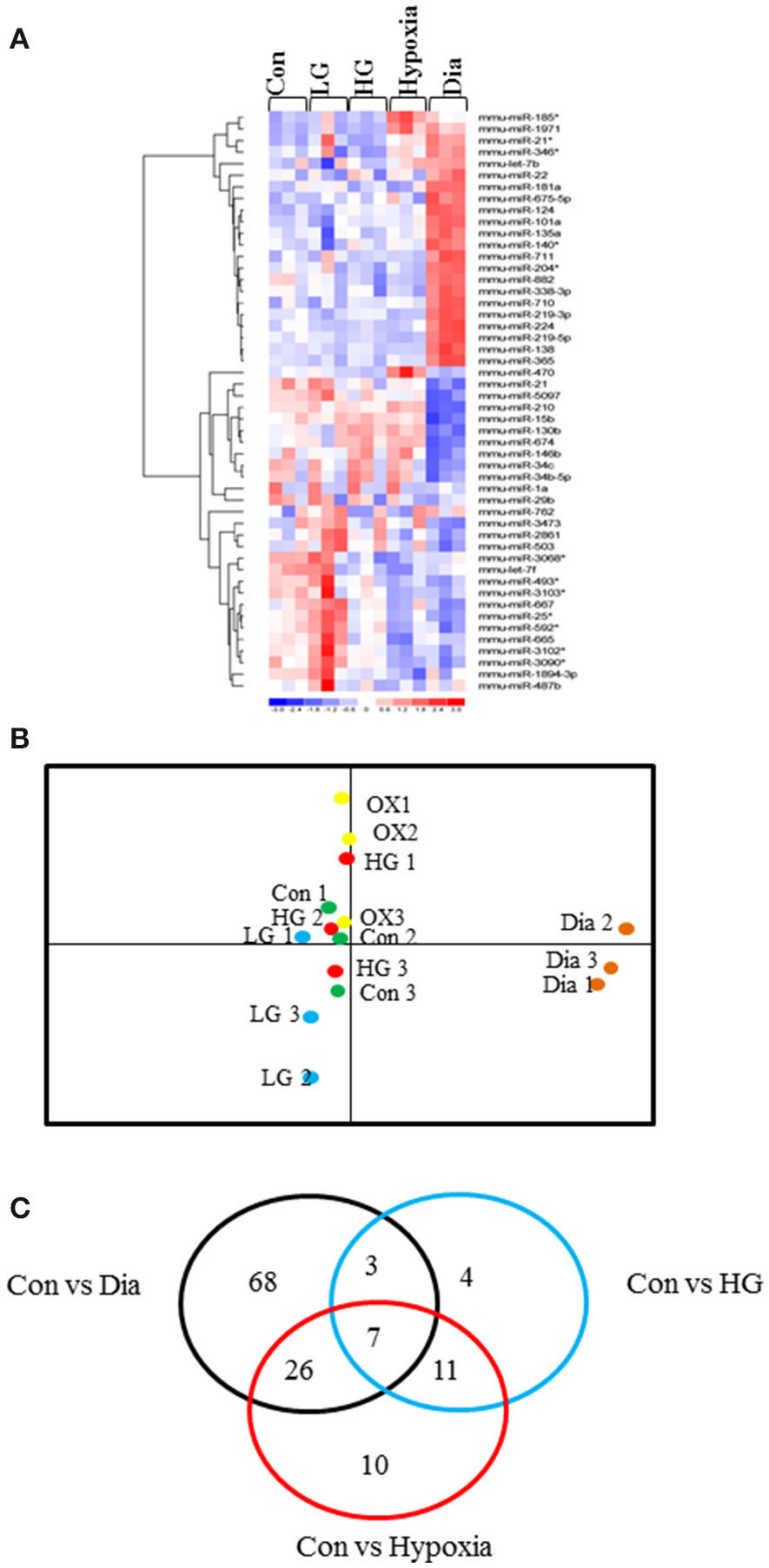
**(A)** Representative heat map shows the top 50 differentially expressed miRNAs in NSCs from control or treated with high glucose or low glucose or hypoxia and diabetic pregnancy. Each row represents a single miRNA and each column represents one sample. Red represents expression levels above the mean (up regulation) while blue represents expression levels below the mean (down regulation). **(B)** Principal Component Analysis (PCA) plot shows the clustering of samples according to their biology. Each group consists of three biological replicates of NSCs from Control, low glucose (LG), high glucose (HG), hypoxia (OX), diabetic pregnancy (Dia). NSCs from diabetic pregnancy (right extreme) show distinct miRNA expression compared to the other groups. **(C)** Venn diagram shows the number of miRNAs commonly deregulated in NSCs from each group and between the groups (fold change < or >1.2 and *p* < 0.05).

Heat map in Figure 1A shows the hierarchical clustering performed on all samples with regard to the top 50 miRNAs with the highest standard deviations (Table 1). Only those miRNAs that were expressed/ detected in all NSC groups were included.

**Table 1:** Top 50 miRNA with highest standard deviation in NSCs from various groups.

**Annotation**	**Standard deviation**
mmu-miR-210	1.116068
mmu-miR-124	0.934708
mmu-miR-1a	0.886978
mmu-miR-219-5p	0.820122
mmu-miR-138	0.718707
mmu-miR-219-3p	0.66549
mmu-miR-882	0.6072
mmu-miR-25^*^	0.58339
mmu-miR-710	0.555703
mmu-miR-762	0.542564
mmu-miR-185^*^	0.539079
mmu-miR-135a	0.537415
mmu-miR-592^*^	0.51364
mmu-miR-204^*^	0.503336
mmu-miR-338-3p	0.499685
mmu-miR-711	0.487714
mmu-miR-21	0.477586
mmu-miR-21^*^	0.473828
mmu-miR-1971	0.473751
mmu-miR-34c	0.452319
mmu-miR-29b	0.439415
mmu-miR-3102^*^	0.433453
mmu-miR-34b-5p	0.431208
mmu-miR-346^*^	0.424621
mmu-miR-5097	0.422695
mmu-miR-470	0.411766
mmu-miR-101a	0.400191
mmu-miR-3068^*^	0.399843
mmu-miR-493^*^	0.388133
mmu-miR-22	0.386176
mmu-miR-365	0.385798
mmu-miR-146b	0.38489
mmu-miR-1894-3p	0.378694
mmu-miR-2861	0.376601
mmu-miR-667	0.362443
mmu-miR-3090^*^	0.361189
mmu-miR-224	0.358282
mmu-miR-181a	0.356957
mmu-miR-665	0.349929
mmu-miR-130b	0.349844
mmu-miR-15b	0.344727
mmu-miR-487b	0.344428
mmu-miR-3103^*^	0.342521
mmu-miR-675-5p	0.335724
mmu-miR-503	0.334091
mmu-miR-3473	0.325719
mmu-miR-140^*^	0.325448
mmu-let-7f	0.324264
mmu-miR-674	0.323366
mmu-let-7b	0.322691

* *Represents the miRNA with minor expression (http://www.mirbase.org/help/nomenclature.shtml)*.

### NSCs from diabetic pregnancy show distinct miRNA expression pattern

Since our study involved several experimental groups, we performed Principal Component Analysis (PCA) on the top 50 miRNAs with highest standard deviations, to identify differences and similarities between the experimental groups. This method was used to trim down large data sets and cluster samples based on their expression profile, which results in separation of samples in different regions of the plot. The PCA analysis revealed that the NSCs from diabetic pregnancy were distinct (based on the expression profile) from the other experimental groups (Figure 1B) and hence were chosen for further analysis. Moreover, the LG group was excluded for further analysis since they did not cluster closely in the PCA plot.

### IPA pathway analysis identified axonal guidance as the top pathway targeted by deregulated MiRNAs

**Figure 2:**
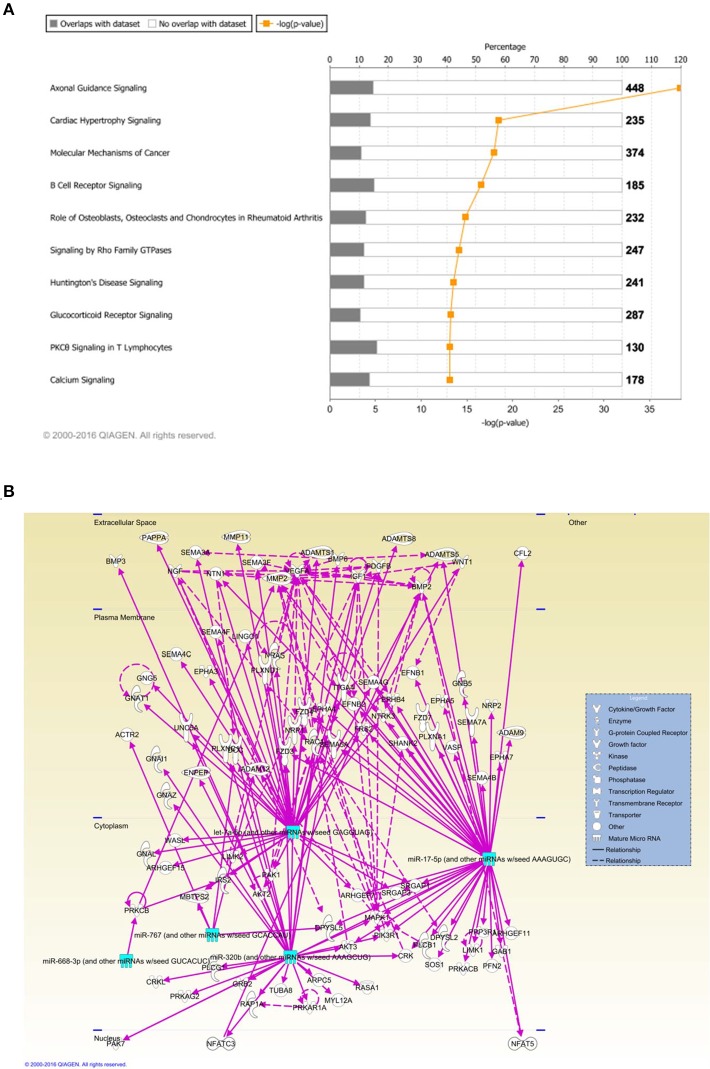
**(A)** Ingenuity pathway analysis (IPA) showed that 5 miRNAs (outlined in cyan) commonly deregulated between NSCs from diabetic pregnancy, hypoxia or high glucose groups could target genes from the axonal guidance pathway in the nervous system. **(B)** Axonal guidance pathway gene targets of the differentially regulated miRNAs. Solid lines represent direct relationship while dotted lines represent indirect relationship.

Ingenuity Pathway Analysis (IPA) was performed to identify biological pathways regulated by differentially expressed miRNAs. miRNAs with a fold change <1.2 or >1.2 and with *p* < 0.05 were selected in each of the NSC groups. A total of 25 miRNAs [Control (Con) vs. HG], 54 miRNAs (Con vs. OX), and 104 miRNAs [Con vs. Diabetic (Dia)] were found to be differentially expressed (Tables 2–4). The number of differentially expressed miRNAs in each group and miRNAs common between the groups are shown in Figure 1C. A comparison revealed that seven miRNAs (let7^*^, let-7a-5p, miR-17^*^, miR-320, miR-668, miR-767, miR3068-3p), were commonly deregulated in NSCs from diabetic pregnancy, hypoxia and HG groups when compared to the control (Table 5). The expression levels of these deregulated miRNAs were similar across the groups (i.e., they were either down regulated or up regulated in all three groups when compared to the control) except for let 7^*^, suggesting that at least six miRNAs had crucial roles in response to glucose or glucose-induced hypoxia. In order to identify the significance of the seven commonly deregulated miRNAs, IPA pathway analysis was carried out. From this analysis, it appeared that majority of the gene targets of five out of seven miRNAs were from the axonal guidance pathway (Figures 2A,B, Table 6).

**Table 2:** miRNAs altered in NSCs exposed to high glucose vs. control.

**ID**	**Fold change**	***p*-value**
mmu-miR-98	−1.253	0.006
mmu-let-7f-1^*^	1.251	0
mmu-let-7f	−1.377	0.011
mmu-miR-99a	−1.281	0.007
mmu-miR-1224^*^	−1.261	0.039
mmu-miR-1247	1.253	0.003
mmu-miR-1249	1.305	0.026
mmu-miR-130b	1.223	0.024
mmu-miR-195	−1.227	0.005
mmu-miR-20b	−1.364	0.011
mmu-miR-187^*^	−1.284	0.004
mmu-miR-1894-3p	−1.216	0.001
mmu-miR-207	−1.311	0.041
mmu-miR-3068^*^	−1.629	0.01
mmu-miR-320	1.273	0.003
mmu-miR-361^*^	1.395	0.009
mmu-miR-693-5p	−1.29	0.001
mmu-miR-669d	1.211	0.018
mmu-miR-500	1.2	0.014
mmu-miR-542-5p	−1.206	0.015
mmu-miR-668	−1.305	0.01
mmu-miR-763	−1.217	0.041
mmu-miR-767	−1.269	0.033
mmu-miR-883a-5p	−1.248	0.018
mmu-miR-883b-5p	−1.217	0.008

* *Represents the miRNA with minor expression (http://www.mirbase.org/help/nomenclature.shtml)*.

**Table 3:** miRNAs altered in NSCs exposed to hypoxia vs. control.

**ID**	**Fold change**	***p*-value**
mmu-let-7e	−1.351	0.002
mmu-let-7b^*^	1.345	0.025
mmu-let-7f	−1.56	0.008
mmu-miR-1224^*^	−1.299	0.019
mmu-miR-1249	1.252	0.025
mmu-miR-125b-2-3p	1.23	0.014
mmu-miR-1264-3p	−1.204	0.011
mmu-miR-138-2^*^	−1.274	0.008
mmu-miR-155^*^	−1.248	0.002
mmu-miR-20b	−1.508	0.006
mmu-miR-185^*^	2.472	0.003
mmu-miR-1894-3p	−1.499	0.011
mmu-miR-191	1.218	0.001
mmu-miR-207	−1.424	0.014
mmu-miR-21^*^	1.443	0.004
mmu-miR-210	1.501	0.021
mmu-miR-210^*^	1.321	0.002
mmu-miR-219-3p	−1.228	0.018
mmu-miR-219-5p	−1.365	0.021
mmu-miR-25^*^	−1.411	0.046
mmu-miR-30d	1.211	0.028
mmu-miR-3068^*^	−1.609	0.004
mmu-miR-3069-5p	1.238	0.005
mmu-miR-3090^*^	−1.396	0.014
mmu-miR-3098-3p	1.269	0.001
mmu-miR-3103^*^	−1.441	0.001
mmu-miR-320	1.222	0.003
mmu-miR-326	−1.301	0.027
mmu-miR-339-5p	1.275	0.005
mmu-miR-361^*^	1.342	0.001
mmu-miR-433^*^	−1.24	0.001
mmu-miR-693-5p	−1.246	0.001
mmu-miR-450a-1^*^	−1.284	0.004
mmu-miR-467g	1.202	0.002
mmu-miR-493^*^	−1.362	0.005
mmu-miR-500	1.261	0.004
mmu-miR-541^*^	−1.233	0.002
mmu-miR-542-5p	−1.24	0.042
mmu-miR-592^*^	−1.471	0.028
mmu-miR-665	−1.373	0.036
mmu-miR-668	−1.309	0.003
mmu-miR-3102^*^	−1.365	0.009
mmu-miR-708	1.228	0.033
mmu-miR-470	1.751	0.018
mmu-miR-744	1.35	0.001
mmu-miR-767	−1.358	0.01
mmu-miR-883b-5p	−1.302	0.003
mmu-miR-421	1.201	0.011
mmu-miR-1900	1.261	0.024
mmu-miR-1971	2.012	0.008
mmu-miR-691	−1.349	0
mmu-miR-695	1.213	0.031
mmu-miR-721	−1.252	0.042
mmu-miR-882	−1.472	0.05

* *Represents the miRNA with minor expression (http://www.mirbase.org/help/nomenclature.shtml)*.

**Table 4:** miRNAs altered in NSCs from diabetic pregnancy vs. control.

**ID**	**Fold change**	***p*-value**
mmu-let-7g	1.398	0.02
mmu-let-7f	−1.327	0.01
mmu-miR-99a	1.257	0.016
mmu-miR-101a	1.879	0
mmu-miR-103	1.216	0.03
mmu-miR-346^*^	1.67	0.001
mmu-miR-124	5.058	0
mmu-miR-125b-2-3p	1.286	0.008
mmu-miR-125a-5p	1.515	0.003
mmu-miR-1264-3p	−1.301	0.001
mmu-miR-128	1.727	0
mmu-miR-130b	−1.504	0.001
mmu-miR-135a	2.131	0.001
mmu-miR-136	1.494	0.023
mmu-miR-138-2^*^	−1.246	0.021
mmu-miR-138	3.164	0.001
mmu-miR-139-5p	−1.306	0.017
mmu-miR-140	1.464	0.003
mmu-miR-140^*^	1.599	0.001
mmu-miR-146b	−1.538	0.038
mmu-miR-15b	−1.682	0.003
mmu-miR-153	1.368	0.003
mmu-miR-195	1.216	0.038
mmu-miR-20b	−1.393	0.005
mmu-miR-181a-1^*^	1.291	0.025
mmu-miR-181a	1.684	0.003
mmu-miR-183^*^	−1.294	0.028
mmu-miR-185	1.227	0.003
mmu-miR-185^*^	1.418	0.025
mmu-miR-187	1.403	0.002
mmu-miR-1897-5p	1.496	0.001
mmu-miR-19a	−1.33	0.045
mmu-miR-1949	−1.46	0.007
mmu-miR-204^*^	1.974	0
mmu-miR-21^*^	1.988	0.001
mmu-miR-21	−2.091	0.004
mmu-miR-210	−5.438	0
mmu-miR-210^*^	−1.243	0.001
mmu-miR-219-3p	2.662	0
mmu-miR-219-5p	3.238	0.001
mmu-miR-22	1.6	0.017
mmu-miR-22^*^	1.356	0.008
mmu-miR-222	−1.252	0.002
mmu-miR-224	1.674	0.003
mmu-miR-23a	−1.221	0.015
mmu-miR-92b	1.493	0.004
mmu-miR-25^*^	−1.727	0.001
mmu-miR-26b	1.443	0.001
mmu-miR-26a	1.661	0
mmu-miR-27a	−1.343	0.013
mmu-miR-291a-3p	−1.36	0.001
mmu-miR-30d	1.517	0.002
mmu-miR-302a	1.37	0.005
mmu-miR-3068	1.509	0
mmu-miR-3068^*^	−1.381	0.005
mmu-miR-3069-3p	−1.227	0.001
mmu-miR-3081^*^	−1.326	0.014
mmu-miR-3084	−1.265	0.002
mmu-miR-3084^*^	−1.289	0.002
mmu-miR-3090^*^	−1.391	0.041
mmu-miR-3103^*^	−1.404	0.004
mmu-miR-320	1.334	0.001
mmu-miR-328	1.342	0.002
mmu-miR-331-3p	1.412	0
mmu-miR-335-5p	1.237	0.001
mmu-miR-338-3p	1.87	0.003
mmu-miR-339-5p	1.271	0.016
mmu-miR-34c	−1.764	0.025
mmu-miR-342-3p	1.45	0.036
mmu-miR-34c^*^	−1.201	0.034
mmu-miR-365	1.817	0.001
mmu-miR-374	−1.245	0.008
mmu-miR-450a-1^*^	−1.288	0
mmu-miR-455	1.254	0.016
mmu-miR-493^*^	−1.474	0.003
mmu-miR-541^*^	−1.343	0.003
mmu-miR-592^*^	−1.6	0.036
mmu-miR-667	−1.388	0.037
mmu-miR-668	−1.364	0.004
mmu-miR-669c^*^	−1.201	0.036
mmu-miR-467b^*^	−1.285	0.009
mmu-miR-674	−1.47	0.001
mmu-miR-674^*^	−1.207	0.005
mmu-miR-1947^*^	1.279	0.023
mmu-miR-675-5p	1.741	0.001
mmu-miR-3102^*^	−1.344	0.029
mmu-miR-708	1.797	0.003
mmu-miR-711	2.099	0.005
mmu-miR-744	1.569	0
mmu-miR-767	−1.555	0.001
mmu-miR-7a	1.299	0.004
mmu-miR-421	−1.218	0.016
mmu-miR-1895	1.583	0.001
mmu-miR-1900	1.288	0.041
mmu-miR-1934	1.681	0.004
mmu-miR-1952	1.237	0.002
mmu-miR-1958	1.334	0.029
mmu-miR-1971	1.738	0.002
mmu-miR-2137	1.723	0.001
mmu-miR-691	−1.203	0.003
mmu-miR-706	1.207	0.039
mmu-miR-710	2.418	0.005
mmu-miR-714	1.436	0.013
mmu-miR-882	1.843	0.013

* *Represents the miRNA with minor expression (http://www.mirbase.org/help/nomenclature.shtml)*.

**Table 5:** miRNAs commonly deregulated in NSCs from diabetic pregnancy, hypoxia and high glucose vs. control.

**Symbol**	**Entrez gene name**	**Dia vs. Con**		**Hg vs. Con**		**Hyp vs. Con**	
		**Exp. *p*-value**	**Exp. fold change**	**Exp. *p*-value**	**Exp. fold change**	**Exp. *p*-value**	**Exp. fold change**
let-7	microRNA let-7a-1	0.02	1.398	0.006	−1.253	0.002	−1.351
let-7a-5p (and other miRNAs w/seed GAGGUAG)	–	0.01	−1.327	0.011	−1.377	0.008	−1.56
miR-17	microRNA 17	0.005	−1.393	0.011	−1.364	0.006	−1.508
miR-320	microRNA 320a	0.001	1.334	0.003	1.273	0.003	1.222
miR-668	microRNA 668	0.004	−1.364	0.01	−1.305	0.003	−1.309
miR-767	microRNA 767	0.001	−1.555	0.033	−1.269	0.01	−1.358
miR-3068-3p (miRNAs w/seed GUGAAUU)	–	0.005	−1.381	0.01	−1.629	0.004	−1.609

**Table 6:** Predicted gene targets from axonal guidance pathway.

**microRNA**	**Gene targets from axonal guidance pathway**
let-7a-5p (and other miRNAs w/seed GAGGUAG) miR-17-5p (and other miRNAs w/seed AAAGUGC) miR-320b (and other miRNAs w/seed AAAGCUG) miR-668-3p (and other miRNAs w/seed GUCACUC) miR-767 (and other miRNAs w/seed GCACCAU)	ACTR2, ADAM12, ADAM9, ADAMTS1, ADAMTS5, ADAMTS8, AKT2, AKT3, ARHGEF11, ARHGEF15, ARHGEF7, ARPC5, BMP2, BMP3, BMP6, CFL2, CRK, CRKL, DCC, DPYSL2, DPYSL5, EFNB1, EFNB2, ENPEP, EPHA3, EPHA4, EPHA5, EPHA7, EPHB4, FRS2, FZD3, FZD4, FZD7, GAB1, GNAI1, GNAL, GNAT1, GNAZ, GNB5, GNG5, GRB2, IGF1, IRS2, ITGA4, LIMK1, LIMK2, LINGO1, MAPK1, MBTPS2, MMP11, MMP2, MYL12A, NFAT5, NFATC3, NGF, NRAS, NRP1, NRP2, NTN1, NTRK3, PAK1, PAK7, PAPPA, PDGFB, PFN2, PIK3R1, PLCB1, PLCG1, PLXNA1, PLXNC1, PLXND1, PPP3R1, PRKACB, PRKAG2, PRKAR1A, PRKCB, RAC1, RAP1A, RASA1, SEMA3A, SEMA3F, SEMA4B, SEMA4C, SEMA4F, SEMA4G, SEMA5A, SEMA7A, SHANK2, SOS1, SRGAP1, SRGAP3, TUBA8, UNC5A, VASP, VEGFA, WASL, WNT1

Furthermore, three miRNAs were common between Con vs. HG and Con vs. Dia groups (Supplementary Table 1), 11 miRNAs were common between Con vs. HG and Con vs. Hypoxia groups (Supplementary Table 2), and 26 miRNAs were common between Con vs. Dia and Con vs. Hypoxia groups (Supplementary Table 3). The expression levels of the majority of miRNAs were similar between the groups compared and large numbers of miRNAs were common between Con vs. Hypoxia and Con vs. Dia groups, suggesting that these miRNAs could be biomarkers of high glucose/hypoxia.

**Figure 3:**
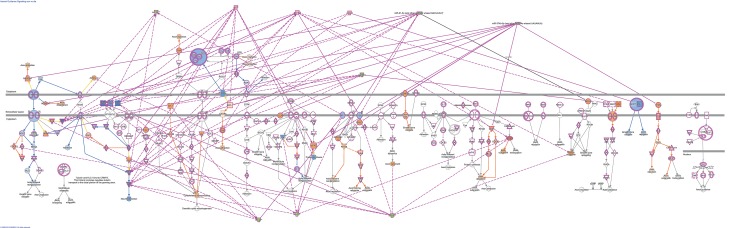
miRNAs altered in NSCs from diabetic pregnancy and their gene targets (outlined in purple) in the axonal guidance pathway. Red represents the upregulated miRNAs while green represents the downregulated miRNAs in NSCs from diabetic pregnancy. Gene predicted to be activated are represented in blue while those inhibited are in blue. Solid lines represent direct relationship while dotted lines represent indirect relationship.

**Figure 4:**
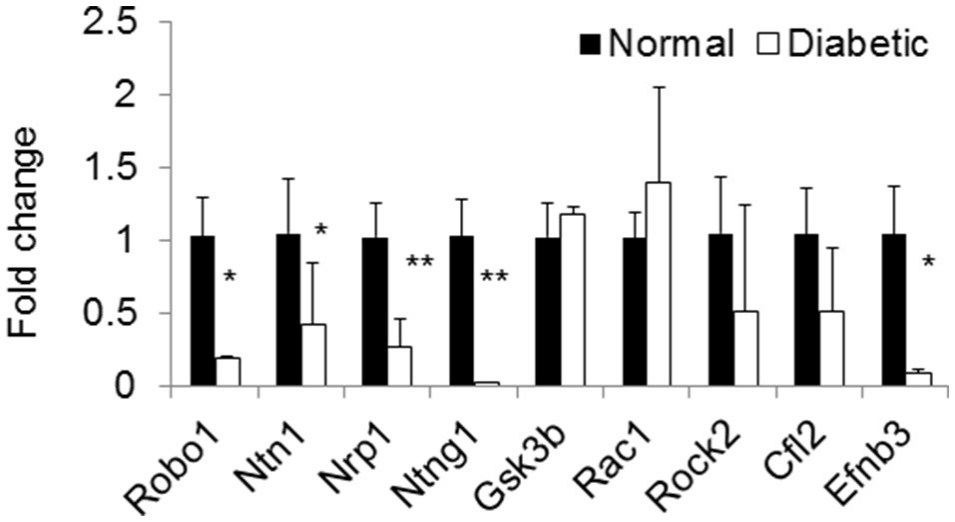
qPCR shows the expression of specific genes related to axon guidance pathway were significantly altered in NSCs from diabetic pregnancy when compared to the control. *p < 0.05, **p < 0.01. β-Actin was used as the control.

Using microRNA target filter, the mRNAs predicted to be targets of the differentially expressed miRNAs were selected and core analysis was performed for each group vs. the control. The top five canonical pathways predicted in each group, signify that maximum miRNAs deregulating that pathway are altered by glucose or glucose-induced hypoxia (Table 7). Comparison analysis revealed that axonal guidance signaling was the top canonical pathway regulated by the differentially expressed miRNAs in NSCs exposed to different conditions (Table 8). miRNAs deregulated in NSCs from diabetic pregnancy and their targets in axonal guidance signaling pathway are represented in Figure 3. The expression of specific genes (*Robo1, Ntn1, Nrp1, Ntng1, Gsk3b, Rac1, Rock2, Clf2, Efnb3*) from this pathway were quantitated by qPCR, and five genes (*Robo1, Ntn1, Nrp1, Ntng1, Efnb3*) were found to be down regulated in NSCs from diabetic pregnancy when compared to the control (Figure 4), suggesting that hyperglycemia deregulates axonal guidance signaling.

**Table 7:** Top five canonical pathways predicted in each NSC group.

**Name**	***p*-value**	**Overlap %**
**CON vs. DIA**		
Axonal guidance signaling	7.46 E-246	63.4
Molecular mechanisms of cancer	1.25E-183	59.9
Huntington's disease signaling	8.66E-133	64.3
Role of macrophages, fibroblasts ad endothelial cells in rheumatoid arthritis	5.57 E-124	53.7
Cardiac hypertrophy signaling	3.46E-120	61.7
**CON vs. HG**		
Axonal guidance signaling	1.07E-91	25.4
Molecular mechanisms of cancer	7.37E-72	24.6
Ephrin receptor signaling	1.09E-50	32.2
Glioblastoma multiforme signaling	2.43E-50	34
Cardiac hypertrophy signaling	3.84E-50	26.4
**CON vs. HYPOXIA**		
Molecular mechanisms of cancer	7.00E-70	24.1
Axonal guidance signaling	1.39E-69	21.4
Wnt/-catenin signaling	7.05E-45	30.2
Cardiac hypertrophy signaling	3.68E-38	22.1
Colorectal cancer metastasis signaling	3.78E-38	21.5

**Table 8:** Comparison of top 30 canonical pathways between NSCs from different groups.

	***p*****-value**		
**Canonical pathway**	**Control vs. hypoxia**	**Control vs. high glucose**	**Control vs. diabetes**
Axonal guidance signaling	68.85794383	90.96895412	245.1269901
Molecular mechanisms of cancer	69.15519592	71.13265984	182.9031945
Cardiac hypertrophy signaling	37.43369777	49.41609122	119.4606899
Huntington's disease signaling	28.98249491	40.06411545	132.0627131
Ephrin receptor signaling	36.88232812	49.96241152	110.6530736
Wnt/β-catenin signaling	44.1516868	38.53985263	109.8974763
Role of macrophages, fibroblasts, and endothelial cells in rheumatoid arthritis	27.13700054	41.64022215	123.2539796
Breast cancer regulation by Stathmin1	35.9936519	39.3877787	111.5120527
Colorectal cancer metastasis signaling	37.42244661	45.47785329	102.4344579
Role of NFAT in cardiac hypertrophy	32.36028786	38.19478616	108.5896027
Glioblastoma multiforme signaling	32.397064	49.61514769	95.40184274
Thrombin signaling	26.72056059	34.51446198	111.5120527
CREB signaling in neurons	27.32224578	36.65050823	102.8422888
Role of osteoblasts, osteoclasts, and chondrocytes in rheumatoid arthritis	28.63946084	39.81557707	95.09929752
ERK/MAPK signaling	27.07227677	34.94309953	92.49876357
IL-8 signaling	25.04059862	38.84921558	90.39369324
Glucocorticoid receptor signaling	24.63806081	43.69089255	83.73600228
CXCR4 signaling	23.4296796	35.19989147	89.64819968
NGF signaling	23.70807143	42.6828916	80.68542543
HGF signaling	26.49634898	40.10672392	80.09516344
Endothelin-1 signaling	27.04021379	33.84264535	85.52064964
Signaling by Rho family GTPases	26.39486393	31.4812889	85.43349271
Phospholipase C signaling	30.40617869	26.91892841	85.70249204
B cell receptor signaling	23.87690481	42.92581301	75.24329644
Gap junction signaling	22.05306461	32.33270321	82.54047604
G-protein coupled receptor signaling	22.80661852	31.74234171	79.5170968
Dopamine-DARPP32 feedback in cAMP signaling	26.00742192	25.80183012	80.49633238
NRF2-mediated oxidative stress response	17.07935798	26.27099868	87.60161075
GNRH signaling	17.76901738	33.56055617	79.45115561
Ovarian cancer signaling	18.6546665	35.40041575	74.41220233

### miRNA-30 family and brain development

**Figure 5:**
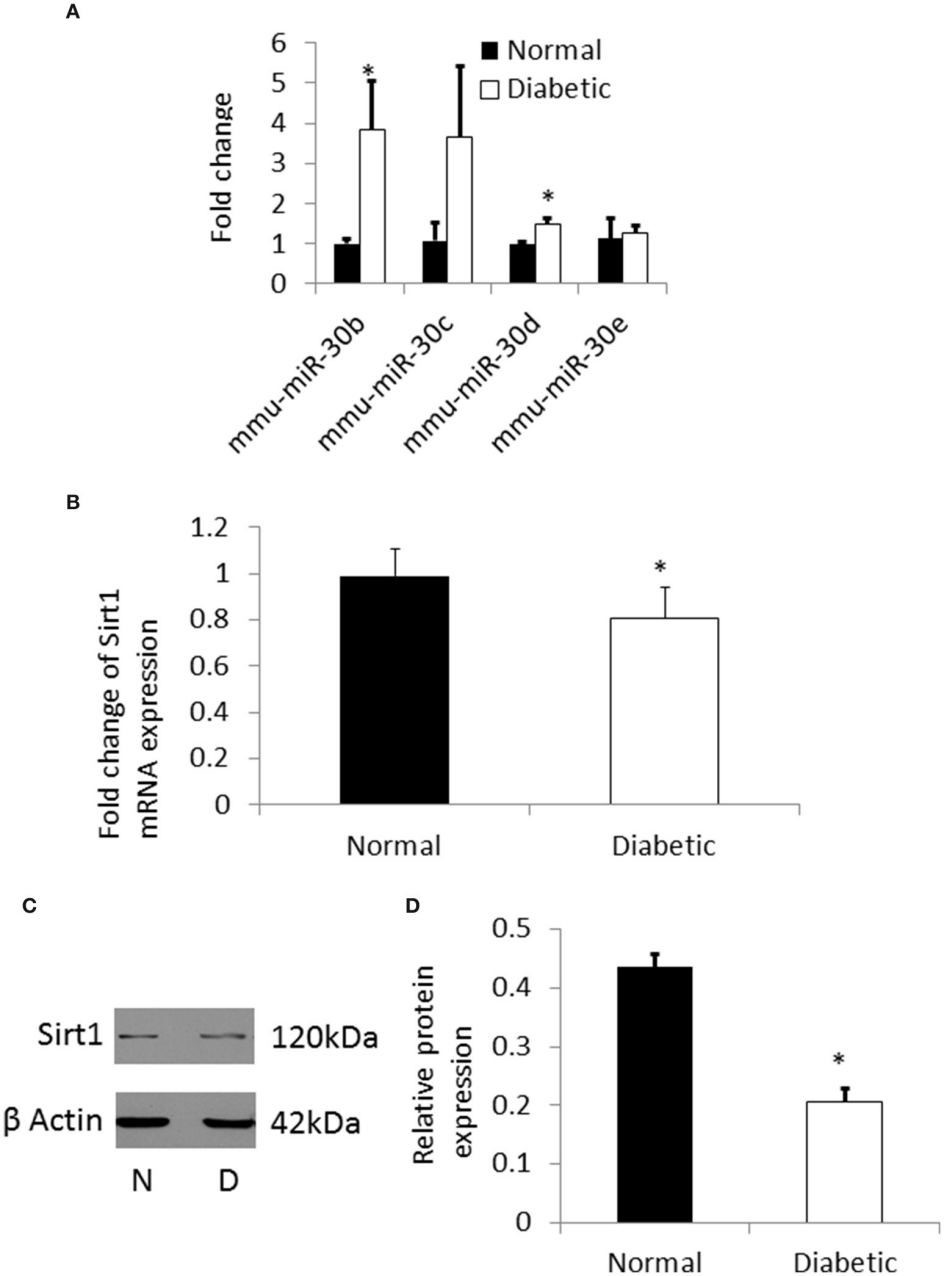
**(A)** qRT-PCR showing the expression pattern of miRNA-30 family. There was significant up regulation of miR-30b and miR-30d in NSCs from diabetic pregnancy (open bars) when compared to normal (black bars). **(B)** qRT-PCR result showing the down regulation of Sirt1 mRNA in NSCs from diabetic pregnancy (open bar) when compared to normal (black bar). **(C)** Representative blots showing the expression of Sirt1 protein in NSCs from diabetic pregnancy and normal. β-Actin was used as the control. **(D)** Bar graph shows significant decrease in Sirt1 protein expression in NSCs from diabetic pregnancy (open bar) when compared to normal (black bar). *p < 0.05.

From the microarray results, a total of 104 miRNAs were found to be differentially expressed in NSCs from embryos of diabetic pregnancy when compared to the control. Among the differentially expressed miRNAs, members of miRNA-30 family (miRNA 30a, b, c, d, and e) were chosen for further studies as they have been found to be: (a) upregulated significantly (fold change ranging from 1.15–1.52) in NSCs from embryos of diabetic pregnancy (Supplementary Table 4); (b) involved in neurodevelopmental disorders (Mellios and Sur, 2012; Hancock et al., 2014; Sun et al., 2014; Han et al., 2015). Further, quantitative RT-PCR analysis was performed to validate the expression levels of miRNA-30 family (miRNA-30 b, c, d, and e) in NSCs from embryos of diabetic and control pregnancy. While miR-30b and miR-30d were significantly up regulated in NSCs from embryos of diabetic pregnancy when compared to the control, miRNA-30c and miRNA-30e showed an increasing trend (Figure 5A). Gene targets of the miRNA-30 family were predicted using IPA (Supplementary Figure 1).

### Sirtuin1 expression is decreased in NSCs from embryos of diabetic pregnancy

From the pathway analysis, Sirtuin1 (*Sirt1*), which is predicted to be one of the targets of miRNA-30b was selected for further analysis as it has been shown to be involved in NSC differentiation and fate determination during brain development (Hisahara et al., 2008; Prozorovski et al., 2008). Quantitative RT-PCR and western blot showed significant down regulation of the *Sirt1* gene and protein expression (Figures 5B–D), respectively, in NSCs from embryos of diabetic pregnant mice when compared to normal pregnant mice.

### Sirtuin1 is a direct target of miR-30b

**Figure 6:**
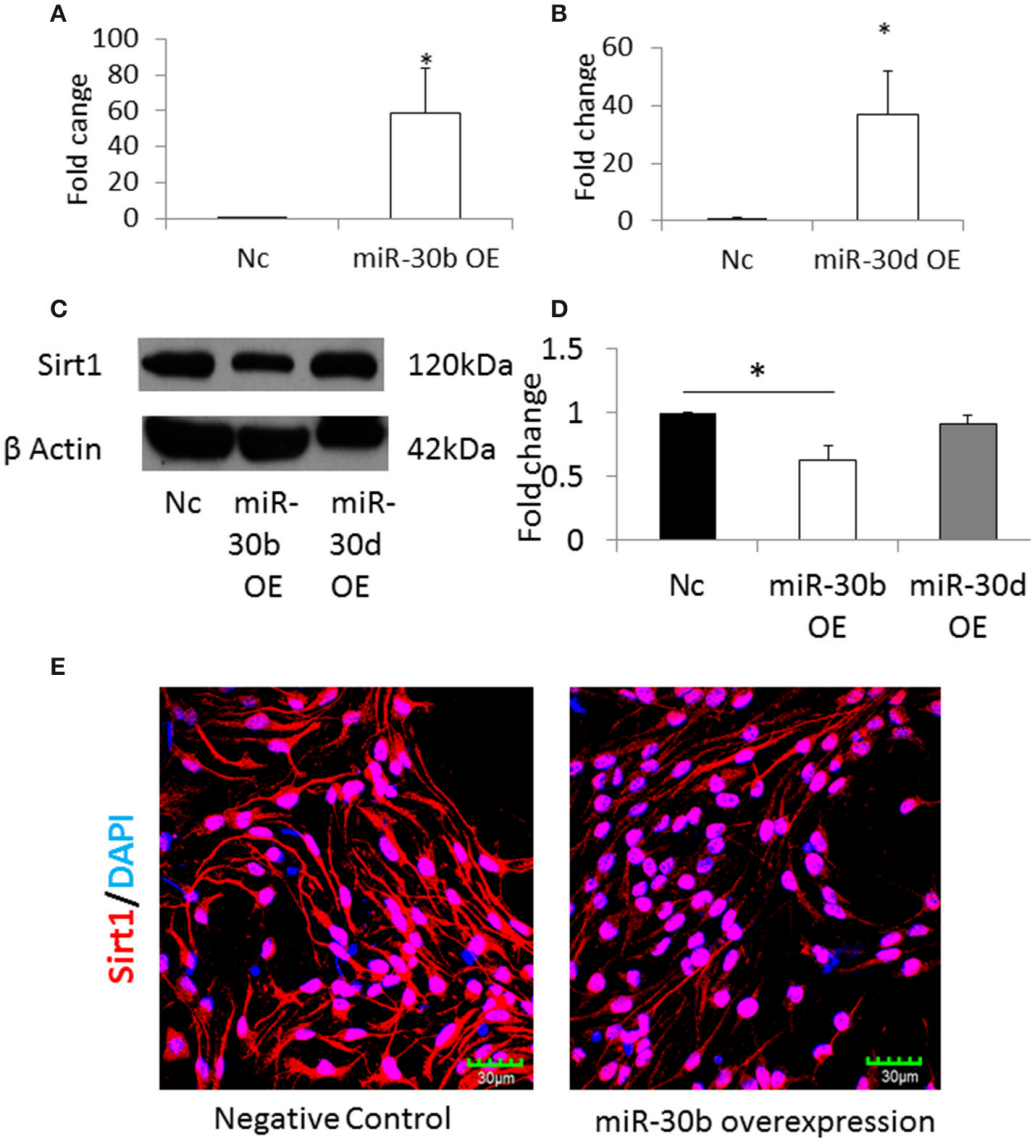
**(A)** qRT-PCR result shows increase in miR-30b expression following transfection with miR-30b mimics. *p = 0.05. **(B)** qRT-PCR result shows increase in expression of miR-30d when NSCs were transfected with miRNA-30d mimics, when compared to negative control. *p = 0.05. **(C)** Representative blot shows the expression of Sirt1 protein in miRNA-30b overexpressed samples when compared to negative control. **(D)** Bar graph showing significant decrease in quantity of Sirt1 protein in miRNA-30b overexpressed NSCs (open bars), slight decrease in quantity of Sirt1 protein in miRNA-30d overexpressed samples (gray bar) when compared to negative control (black bar). *p < 0.05. **(E)** Confocal images showing the expression of Sirt1 in negative control (left) and miRNA-30b mimic (right) transfected NSCs.

The NSCs from normal pregnancy were transfected with miRNA-30b or miRNA-30d mimics, in order to overexpress these miRNAs. Scrambled nucleic acids were transfected in NSCs from normal pregnancy and they were used as the negative control. Following transfection, the expression of miRNA-30b and miRNA-30d increased 60-fold (Figure 6A) and 40-fold (Figure 6B), respectively, when compared to negative control. Western blot analysis revealed that overexpression of miRNA-30b but not miRNA-30d could significantly decrease the expression of Sirt1 protein suggesting that Sirt1 may be a target of miRNA-30b (Figures 6C,D). In addition, immunostaining and confocal imaging following miR-30b overexpression revealed decreased expression of Sirt1 in miRNA-30b overexpressed cells when compared to negative control (Figure 6E).

**Figure 7:**
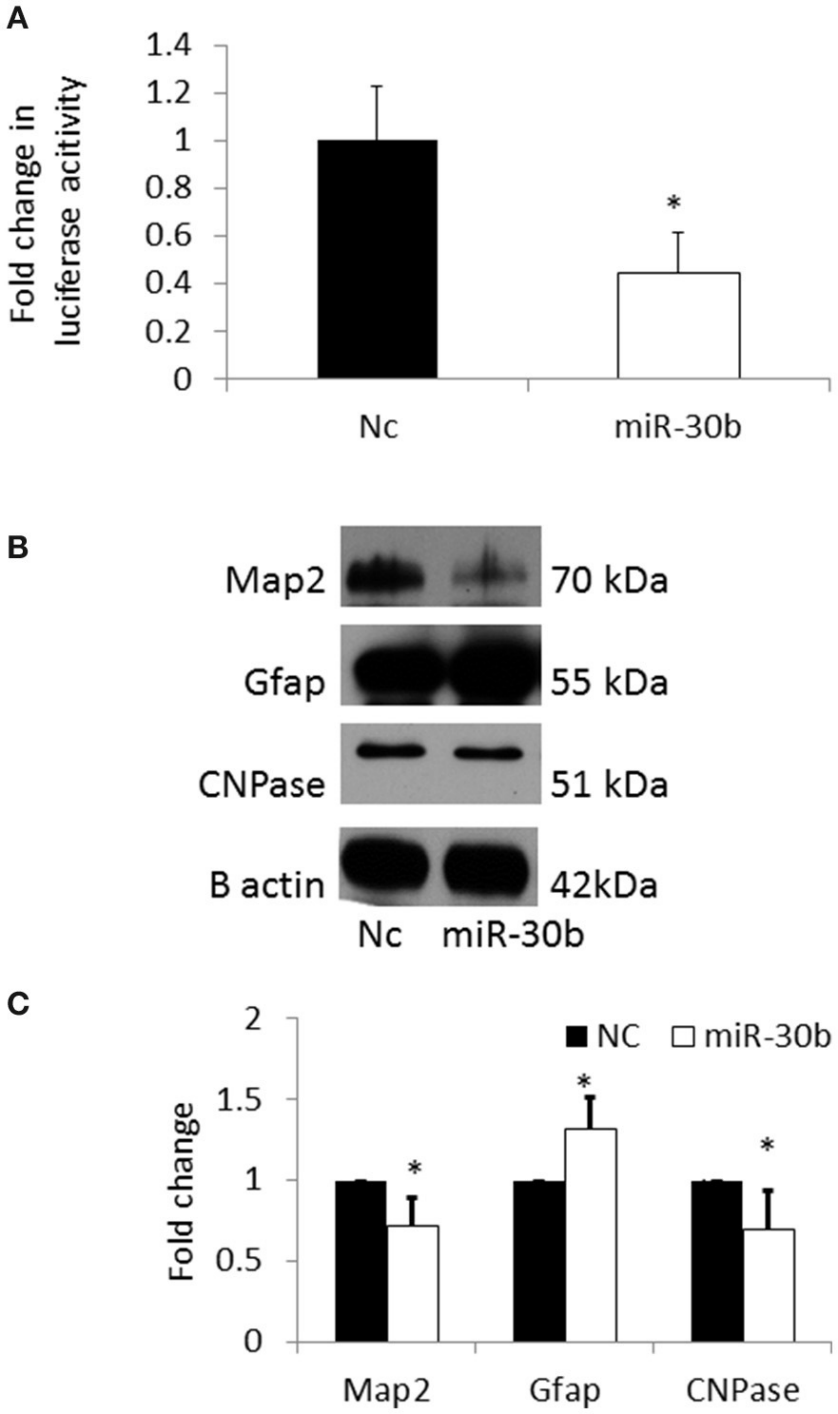
**(A)** Bar graph showing significant decrease in luciferase activity in miRNA-30b mimic and Sirt1 3′UTR plasmid co-transfected BV2 cells (open bar) when compared to the negative control (black bar). **(B)** Representative blots showing the expression of Map2 (neuronal marker) and CNPase (early oligodendrocyte marker), and Gfap (astrocyte marker) proteins in miRNA-30b mimic or scrambled negative control transfected NSCs. β-Actin was used as the control. NC-negative control, miRNA-30b OE—miRNA-30b over-expression. **(C)** Bar graph shows significant decrease in expression of Map2, CNPase proteins and significant increase in expression of Gfap protein in miRNA-30b overexpressed samples (open bars) when compared to negative control (black bars). *p < 0.05. NC, negative control; miRNA-30b OE, miRNA-30b over expression; miRNA-30d OE, miRNA-30d over expression.

In order to confirm that Sirt1 is a direct target of miR-30b, a 3′UTR plasmid assay was performed. *Sirt1* plasmid containing the 3′UTR of *Sirt1* gene was co-transfected with miRNA-30b mimic or negative control mimic in BV2 cells. Following transfection, the luminescence intensity was found to be significantly decreased in miRNA-30b mimic transfected cells (Figure 7A) suggesting that miRNA-30b binds to its complementary site in the 3′UTR of *Sirt1* and directly regulates the expression of Sirt1.

### miRNA-30b alters the fate specification of NSCs via down regulation of Sirt1

The expression levels of neuronal and glial markers were determined following overexpression of miRNA-30b in NSCs in order to understand the role of miRNA-30b on NSC fate specification. Overexpression of miRNA-30b resulted in significant decrease in the expression of MAP2 (neuronal marker) and CNPase (early oligodendrocyte marker) proteins and increase in the expression of GFAP (astrocyte marker) protein when compared to that in negative control (Figures 7B,C).

**Figure 8:**
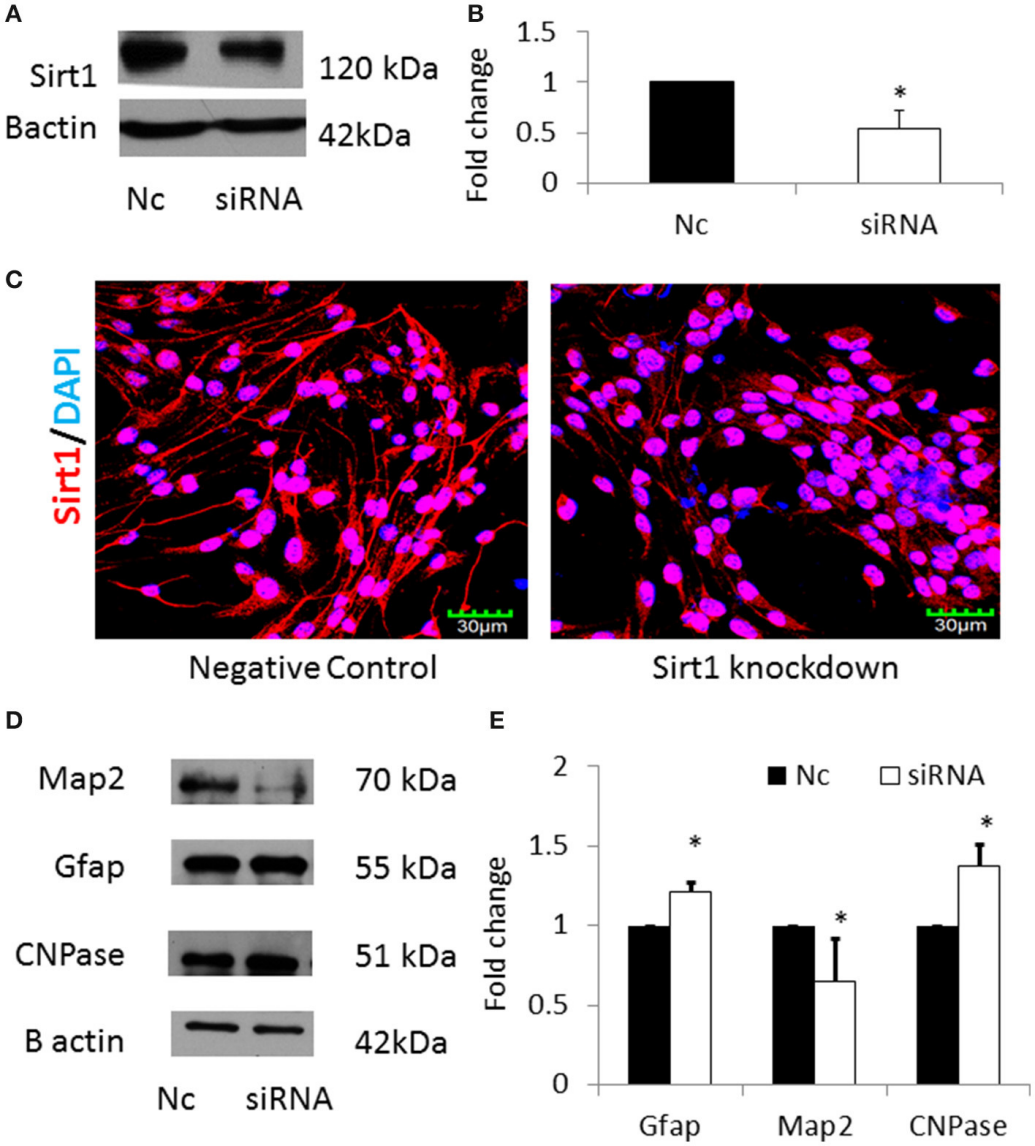
**(A)** Representative blot showing significant down regulation of Sirt1 protein in NSCs transfected with Sirt1 siRNA when compared to negative control. **(B)** Bar graph showing significant decrease in Sirt1 protein in Sirt1 siRNA treated NSCs (open bar) when compared to negative control (black bar). *p < 0.05. **(C)** Confocal images showing expression of Sirt1 following siRNA mediated knockdown of Sirt1 in NSCs. **(D)** Representative blot showing expression of Map2, Gfap and CNPase protein in Sirt1 silenced NSCs when compared to negative control. **(E)** Bar graph showing a significant decrease in Map2 protein and significant increase in Gfap and CNPase proteins in Sirt1 silenced NSCs (open bars) when compared to negative control (black bars). *p < 0.05.

In order to verify that miRNA-30b increased astrocytes via down regulating its specific target Sirt1, Sirt1 was silenced using siRNA in NSCs and the expression of MAP2, GFAP, and CNPase proteins were analyzed. Sirt1 silencing efficiency was found to be 40% (Figures 8A,B). In addition, immunostaining and confocal imaging confirmed decreased Sirt1 expression after siRNA mediated *Sirt1* silencing (Figure 8C). siRNA-mediated knockdown of Sirt1 in NSCs led to an increase in GFAP with a concomitant decrease in MAP2 proteins similar to miRNA-30b overexpression (Figures 8D,E). However, knockdown of Sirt1 also increased the expression of CNPase (in contrast to miRNA-30b overexpression).

## Discussion

The fact that the developing brain is sensitive to glucose, is evident from the substantial incidence of NTDs caused by maternal diabetes (Becerra et al., 1990; Sheffield et al., 2002). In humans, although management of diabetes during pregnancy has limited the number of NTDs to a great extent, there is still concern as infants of diabetic mothers have been shown to develop neuropsychological deficits later in life (ter Braak et al., 2002). Neural tube closure requires complex orchestration of cellular and molecular events, while normal brain function depends on appropriate neuronal wiring (Ayala et al., 2007). Analyzing phenotypically normal embryos from diabetic mothers may therefore be essential in understanding the molecular details, behind the occurrence of altered brain functions in offspring of diabetic mothers. Hence in this study, NSCs were isolated from non-malformed embryos from diabetic and control pregnant mice. To our knowledge, this study is the first of its kind, in profiling miRNA expression in NSCs exposed to hyperglycemia *in vivo*. Furthermore, the *in vitro* groups (high glucose, low glucose and hypoxia group) serve as an excellent comparison as they reflect *in vivo* environment. Highest numbers of deregulated miRNAs were found to be common between NSCs exposed to hyperglycemia and hypoxia, suggesting that glucose-induced oxidative stress and hypoxia may underlie the changes seen in miRNA expression. Such high throughput analysis combined with pathway analysis provides valuable insights into the mechanism of high glucose-induced defects.

Complex processes such as neuronal migration and axon guidance are required for proper wiring in the brain (Marin et al., 2010) and these events require molecules that are involved in cytoskeleton rearrangement and secondly those that act as guidance cues (Uher and Golden, 2000). From the pathway analysis in this study, axonal guidance pathway was found to be the top pathway that was targeted by deregulated miRNAs in NSCs from different groups. The axon guidance pathway encompasses four major ligands (netrin, ephrin, semaphoring, and slit), their receptors (Dcc/unc, neuropilin, plexin, robo, etc.), and other proteins that serve as attraction or repulsion cues in order to mediate axons to their target (Lin et al., 2009). Decreased expression of axon guidance pathway genes, *Robo1, Ntn1, Ntng1, Nrp1*, and *Efnb3* in NSCs exposed to hyperglycemia was suggestive that miRNAs could target and deregulate the axonal guidance pathway as predicted by the pathway analysis. However, further studies are required to ascertain the effect of decreased expression of specific genes from the axonal guidance pathway in inducing alterations in brain development/function.

miRNA-30 family is found to have diverse functions in the brain during development and disease, with well-known roles in regulating epithelial-to-mesenchymal transition (EMT) (Kumarswamy et al., 2012). Members of the miRNA-30 family i.e., miRNA-30a and miRNA-30d, are enriched in layer III pyramidal neurons and have been shown to target BDNF during development (Mellios and Sur, 2012). In addition, miRNA-30d expression levels are found to be affected in brains of female schizophrenic patients (Mellios and Sur, 2012), thus emphasizing the importance of miRNA-30 family in brain development and disease.

In the present study, miRNA-30 family was found to be up regulated in NSCs from diabetic pregnancy when compared to control, suggesting that maternal diabetes alters the expression of miRNA-30 family and its target genes, which may perturb brain development in offspring of diabetic mothers.

One of the miRNA-30 family members, miRNA-30b is found to target Sirt1 which belongs to the Sirtuin family of proteins with seven members of the family being reported to exist in mammals. Sirt1 is the closest analog of yeast Sir2 (Sack and Finkel, 2012) which is highly expressed in fetal brain (Sakamoto et al., 2004) and has extensive role in NSC differentiation and fate determination during brain development (Hisahara et al., 2008; Prozorovski et al., 2008). In addition, Sirt1 is expressed in axonal growth cone wherein it regulates formation and elongation of axons (Li et al., 2013). Studies have shown that Sirt1 knockdown results in neural defects such as exencephaly in mice (Cheng et al., 2003; McBurney et al., 2003) and NTDs in Xenopus embryos (Ohata et al., 2014), suggesting the importance of Sirt1 in neural tube formation. In addition, Sirt1 is known to regulate memory and plasticity (Gao et al., 2010) and is required for normal cognitive function (Michan et al., 2010). Furthermore, absence of Sirt1 led to cognitive disabilities in offspring, such as deficits in spatial learning and classical conditioning (Michan et al., 2010). Recently, it has been shown that Sirt2 and Sirt6 are involved in diabetes-induced NTDs (Yu et al., 2016). Our results show the upregulation of miRNA-30b and downregulation of its target gene, Sirt1 in NSCs derived from non-malformed embryos from diabetic mice which suggest that cognitive deficiency or neuropsychological deficits that are said to be associated with maternal diabetes (in the absence of anatomical anomaly) may be mediated by Sirt1 downregulation. However, it warrants further investigation.

Differentiation of NSCs, the progenitor cells of the nervous system into astrocytes, neurons and oligodendrocytes is a complex process which is modulated by a dynamic molecular web consisting of transcriptional factors and signaling pathways (Wen et al., 2009). Furthermore, the role of epigenetic mechanisms including DNA methylation, histone modifications, and non-coding RNAs, in regulating differentiation or fate determination of NSCs has become imperative. Among the other miRNAs, our results highlight that miRNA-30b controls NSC fate determination via Sirt1, since Sirt1 has been shown to be involved in the final fate specification of NSCs (Cai et al., 2016). Further in particular, overexpression of miRNA-30b and decreased expression of Sirt1 favored the differentiation of NSCs to astrocytes at the expense of neurons. On the other hand, Sirt1 knockdown alone increased the expression of GFAP, an astrocyte marker and CNPase, an oligodendrocyte marker in NSCs suggesting that multiple miRNAs and their gene targets are involved in deciding NSC fate specification.

## Conclusion

Overall, the present data revealed that high glucose/hyperglycemia or hypoxia deregulates the expression of several miRNAs that target crucial gene pathways, such as axonal guidance pathway in NSCs. miRNA microarray coupled with pathway analyses have provided novel insights into the mechanism of hyperglycemia-induced malformations in the brain. In addition, we found that hyperglycemia increased the expression of miRNA-30 family, in particular miRNA-30b that altered NSC differentiation via down regulation of its target, Sirt1 in NSCs.

## Ethics statement

The protocol was reviewed and approved by Office of safety, health and environment (OSHE), National University of Singapore. The procedures pertaining to animal usage was performed in accordance with the guidelines laid by Institutional Animal Care and Use Committee (IACUC), National University of Singapore.

## Author contributions

Study concept and design: SD, SS, and SR. Acquisition, analysis, or interpretation of data: SR, SS, and SD. Drafting of the manuscript: SR, SS, SD, and BB. Critical revision of the manuscript for important intellectual content: SD, BB, SR, and SS. Obtained funding: SD. Study supervision: SD. All authors read and approved the final manuscript.

### Conflict of interest statement

The authors declare that the research was conducted in the absence of any commercial or financial relationships that could be construed as a potential conflict of interest.

## Supplementary material

Supplementary Figure 1 Gene targets of miR-30 family are depicted. Sirt1 and its interacting proteins are highlighted in blue.

Supplementary Table 1 miRNAs common between HG vs. Con, Dia vs. Con groups.

Supplementary Table 2 miRNAs common between HG vs. Con, Hypoxia vs. Con groups.

Supplementary Table 3 miRNAs common between Dia vs. Con, Hypoxia vs. Con groups.

Supplementary Table 4 Fold change and *p*-value of miRNA-30 family.
