# Supplementary figures and images for: Maternal Diabetes Alters Expression of MicroRNAs that Regulate Genes Critical for Neural Tube Development
Source: Front Mol Neurosci. 2017 Jul 27;10:237. doi: 10.3389/fnmol.2017.00237 (PMC5531003; doi:10.3389/fnmol.2017.00237)

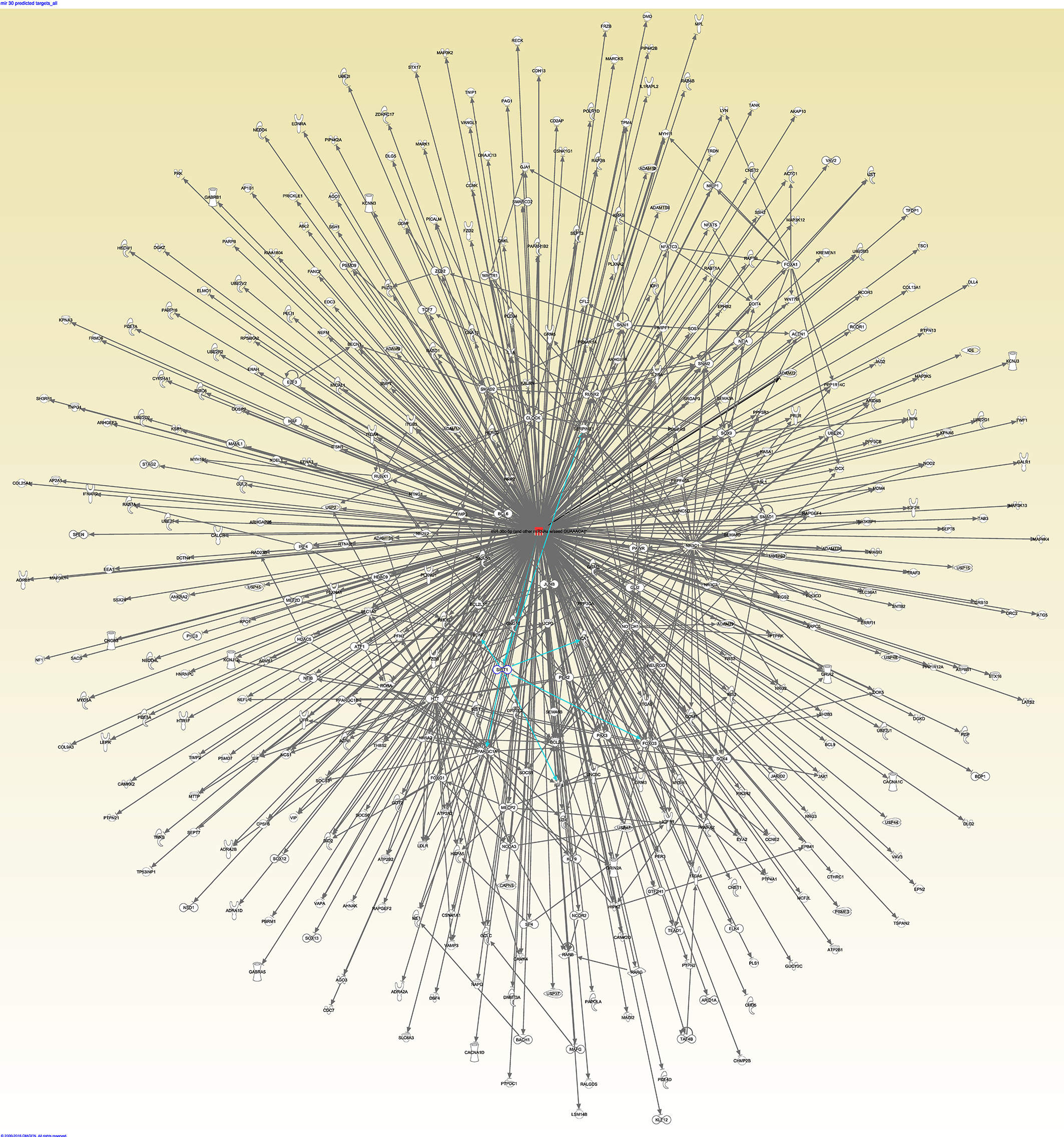

Supplement: Supplementary Figure 1 — Gene targets of miR-30 family are depicted. Sirt1 and its interacting proteins are highlighted in blue. [file Image1.TIFF]
