# Supplementary material for: Maternal Diabetes Alters Expression of MicroRNAs that Regulate Genes Critical for Neural Tube Development
Source: Front Mol Neurosci. 2017 Jul 27;10:237. doi: 10.3389/fnmol.2017.00237 (PMC5531003; doi:10.3389/fnmol.2017.00237)
Supplement: Supplementary Table 1 — miRNAs common between HG vs. Con, Dia vs. Con groups. [file Table1.docx]

Supplementary Material

**Maternal diabetes alters expression of microRNAs that regulate genes critical for neural tube development**

Ramya S^1*^, Sukanya S^1*^, Bay BH^1^, and Dheen ST^1^#

*equal contribution

^1^ Department of Anatomy, Yong Loo Lin School of Medicine, National University Health System, National University of Singapore, Singapore 117597.

**Corresponding author:**

#S.T Dheen

Department of Anatomy

Yong Loo Lin School of Medicine

National University of Singapore

Blk MD10, 4 Medical Drive,

Singapore117597, Singapore

Email: [antstd@nus.edu.sg](mailto:antstd@nus.edu.sg)

Tel: +65-6516-3217: Fax: +65-6778-7643

# Supplementary table 1: miRNAs common between HG vs con and dia vs con groups

|  |  | HG vs con | | Dia vs con | |
| --- | --- | --- | --- | --- | --- |
| Symbol | Entrez Gene Name | Exp p-value | Exp Fold Change | Exp p-value | Exp Fold Change |
| miR-10* | microRNA 100 | 0.007 | -1.281 | 0.016 | 1.257 |
| miR-130* | microRNA 130a | 0.024 | 1.223 | 0.001 | -1.504 |
| mir-16-5p | - | 0.005 | -1.227 | 0.038 | 1.216 |

# Supplementary table 2: miRNAs common between HG vs con and hypoxia vs con groups

|  |  | HG vs con | | Hypoxia vs con | |
| --- | --- | --- | --- | --- | --- |
| Symbol | Entrez Gene Name | Exp p-value | Exp Fold Change | Exp p-value | Exp Fold Change |
| **let-7a-3p** (and other miRNAs w/seed UAUACAA) | -- | 0 | 1.251 | 0.025 | 1.345 |
| mir-207 | microRNA 207 | 0.041 | -1.311 | 0.014 | -1.424 |
| **mir-467** | microRNA 466 | 0.018 | 1.211 | 0.002 | 1.202 |
| **mir-500** | microRNA 501 | 0.014 | 1.2 | 0.004 | 1.261 |
| **mir-1249** | microRNA 1249 | 0.026 | 1.305 | 0.025 | 1.252 |
| miR-1224-3p (miRNAs w/seed CCCACCU) | -- | 0.039 | -1.261 | 0.019 | -1.299 |
| miR-1894-3p (miRNAs w/seed CAAGGGA) | -- | 0.001 | -1.216 | 0.011 | -1.499 |
| **miR-361-3p** (miRNAs w/seed CCCCCAG) | -- | 0.009 | 1.395 | 0.001 | 1.342 |
| miR-449b-3p (and other miRNAs w/seed AGCCACA) | -- | 0.001 | -1.29 | 0.001 | -1.246 |
| miR-542-5p (and other miRNAs w/seed UCGGGGA) | -- | 0.015 | -1.206 | 0.042 | -1.24 |
| miR-883b-5p (miRNAs w/seed ACUGAGA) | -- | 0.008 | -1.217 | 0.003 | -1.302 |

# Supplementary table 3: miRNAs common between dia vs con and hypoxia vs con groups

|  |  | Dia vs con | | Hypoxia vs Con | |
| --- | --- | --- | --- | --- | --- |
| Symbol | Entrez Gene Name | Exp p-value | Exp Fold Change | Exp p-value | Exp Fold Change |
| Mir691 | microRNA 691 | 0.003 | -1.203 | 0 | -1.349 |
| Mir882 | microRNA 882 | 0.013 | 1.843 | 0.05 | -1.472 |
| **Mir1900** | microRNA 1900 | 0.041 | 1.288 | 0.024 | 1.261 |
| **Mir1971** | microRNA 1971 | 0.002 | 1.738 | 0.008 | 2.012 |
| **mir-30** | microRNA 30a | 0.002 | 1.517 | 0.028 | 1.211 |
| mir-95 | microRNA 95 | 0.016 | -1.218 | 0.011 | 1.201 |
| mir-210 | microRNA 210 | 0 | -5.438 | 0.021 | 1.501 |
| **mir-708** | microRNA 708 | 0.003 | 1.797 | 0.033 | 1.228 |
| **mir-744** | microRNA 744 | 0 | 1.569 | 0.001 | 1.35 |
| **miR-125b-2-3p** (miRNAs w/seed CAAGUCA) | -- | 0.008 | 1.286 | 0.014 | 1.23 |
| miR-1264-3p (miRNAs w/seed AAAUCUU) | -- | 0.001 | -1.301 | 0.011 | -1.204 |
| miR-138-2-3p (miRNAs w/seed CUAUUUC) | -- | 0.021 | -1.246 | 0.008 | -1.274 |
| **miR-185-3p** (and other miRNAs w/seed GGGGCUG) | -- | 0.025 | 1.418 | 0.003 | 2.472 |
| **miR-21-3p** (and other miRNAs w/seed AACAGCA) | -- | 0.001 | 1.988 | 0.004 | 1.443 |
| miR-210-5p (and other miRNAs w/seed GCCACUG) | -- | 0.001 | -1.243 | 0.002 | 1.321 |
| miR-219a-2-3p (miRNAs w/seed GAAUUGU) | -- | 0 | 2.662 | 0.018 | -1.228 |
| miR-219a-5p (and other miRNAs w/seed GAUUGUC) | -- | 0.001 | 3.238 | 0.021 | -1.365 |
| miR-25-5p (miRNAs w/seed GGCGGAG) | -- | 0.001 | -1.727 | 0.046 | -1.411 |
| miR-3090-5p (miRNAs w/seed UCUGGGU) | -- | 0.041 | -1.391 | 0.014 | -1.396 |
| miR-3103-5p (and other miRNAs w/seed GAGGGAG) | -- | 0.004 | -1.404 | 0.001 | -1.441 |
| **miR-339-5p** (and other miRNAs w/seed CCCUGUC) | -- | 0.016 | 1.271 | 0.005 | 1.275 |
| miR-450a-1-3p (and other miRNAs w/seed UUGGGAA) | -- | 0 | -1.288 | 0.004 | -1.284 |
| miR-493-5p (miRNAs w/seed UGUACAU) | -- | 0.003 | -1.474 | 0.005 | -1.362 |
| miR-541-3p (miRNAs w/seed GGCGAAC) | -- | 0.003 | -1.343 | 0.002 | -1.233 |
| miR-592-3p (miRNAs w/seed CAUCACG) | -- | 0.036 | -1.6 | 0.028 | -1.471 |
| miR-702-5p (and other miRNAs w/seed UGAGUGG) | -- | 0.029 | -1.344 | 0.009 | -1.365 |

**Supplementary table 4: Fold change and p value of miRNA-30 family**

| microRNA | Fold change (Dia vs control) | P value |
| --- | --- | --- |
| miR 30a | 1.25 | 8.53E-03 |
| miR-30b | 1.17 | 2.54E-02 |
| miR-30c | 1.16 | 1.98E-01 |
| miR-30d | 1.52 | 1.56E-03 |
| miR-30e | 1.15 | 6.21E-02 |

**
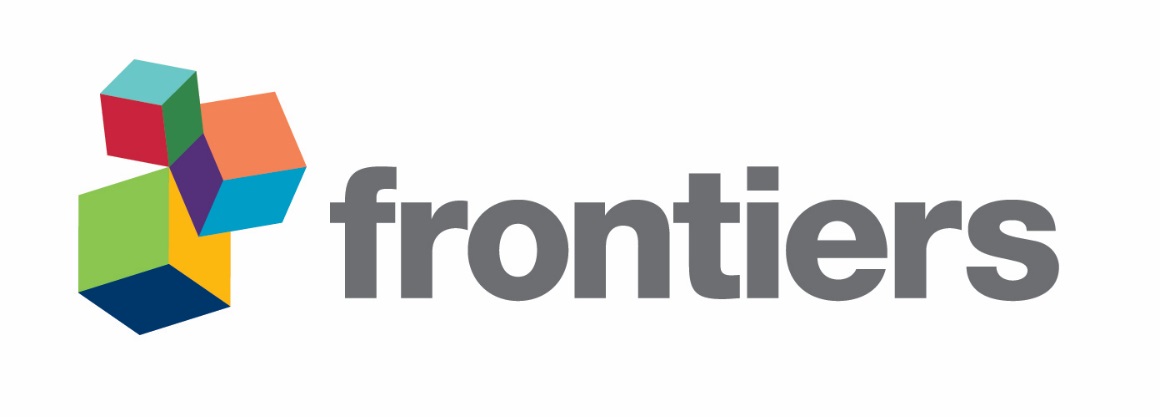
**
